# The Effect of Silver Nanoparticles on Wounds Contaminated with *Pseudomonas aeruginosa* in Mice: An Experimental Study

**Published:** 2017

**Authors:** Malahat Ahmadi, Masood Adibhesami

**Affiliations:** *Associate Professor of Microbiology, Department of Microbiology, Faculty of Veterinary Medicine, Urmia University, Urmia, Iran. *

**Keywords:** Wound infection, *Pseudomonas aeroginosa*, Silver nanoparticles, Mice

## Abstract

The microorganisms have been usually noted as the major cause of delayed wound healing.* Pseudomonas aeruginosa* is the most common pathogen causing these infections. Silver nanoparticles (AgNPs) show ample antibacterial activities. In present study, the effect of AgNPs alone and in combination with tetracycline investigated on inoculated wounds with *Pseudomonas aeruginosa* in mice. Twenty mice anesthetized and full-thickness skin wounds created on back of them and the bacterial suspension added to each wound bed. Wound infection assessed using total count of bacterial load and also wound healing monitored, macroscopically. In all groups treatments applied topically in the wound bed: AgNPs, tetracycline, AgNPs along with tetracycline and normal saline in control group. The tetracycline along with AgNPs achieved 100% wound closure on day 12. In the AgNPs group, the percentage of wound contraction has close figures compared to tetracycline and normal saline as 98, 99 and 79 percent, respectively. By day 12, all of the treated groups with AgNPs, tetracycline and AgNPs along with tetracycline showed decreases in surface bacterial concentration compared with control group. Also, significant decrease (P < 0.001) in deep skin bacterial counts in the AgNPs, tetracycline and AgNPs along with tetracycline compared with control group at any time point. Application of AgNPs along with tetracycline is more effective than AgNPs and tetracycline alone to reduce the bacterial load whilst wound macroscopic contraction increased. These findings support use of the AgNPs in combination with antibacterial medicine for the treatment of infectious skin wounds.

## Introduction

One of the main objectives in wound healing is restoration in the shortest time with minimal side effects. Patients with these infections suffer from pain, loss of functional ability, decreased quality of life and may die (1). Wound infections intricate by pathogen bacteria, and they are important to note that constantly rates of multiantibiotic resistance among these bacteria are increasing today (2). For these reasons, wound site infections have become a problem for patients and health services. Consequently, immediate monitoring of wound infections and preventive control and therapeutic policy have been proposed (3). 

The negative effect of certain types of microorganisms on wound healing has been widely published; the microorganisms have been usually noted as the cause of delayed wound healing. One the most common pathogen causing these infections is* Pseudomonas aeruginosa* (4). Antimicrobial therapy that controls colonization and proliferation of microbial pathogens is the most vital aspects of skin wound care (5). So, antimicrobial medicines, such as silver compounds, iodine compounds, acetic acid, and chlorhexidine are often used to treat or prevent wound infections (6-7). An ideal treatment should be able to protect the wounds against microbial interactions (8). For centuries metals have been applied as bactericidal and bacteriostatic agents. Silver, gold and zinc with different properties and spectrums of activity have been used (9). Nanoparticles are defined as particulate dispersions or solid particles with a size in the range of 1-100 nm (10). The alteration from microparticles to nanoparticles (<100 nm in diameter) includes an increase in relation to the surface area, among other changes in properties. The antibacterial activity of metals depends on their contact surface; a larger surface area of the nanoparticles allows a larger extent of interactions with other organic and inorganic molecules (11). 

Nanomedicine is a developing field expanding rapidly because of the development of new nanomaterial into a range of products and technologies. Silver nanoparticles (AgNPs) show both unique physicochemical properties (high ratio of surface area to mass) and ample antibacterial activities, which confer them as a major advantage for the development of alternative products against, for example, multidrug resistant microorganisms (12) and *in-vitro* condition AgNPs were effective against Gram-negative such as *Pseudomonas aeruginosa* (13). AgNPs are attractive because they are non-toxic to the human body at low concentrations and have broad spectrum antibacterial actions (14). In recent years, there has been great progress in the application of nanoparticles in biological technology. The medicine application of NPs has increased and expanded to the fields of non-infection wound healing (15). In this study, the effect of silver nanoparticles alone and in combination with tetracycline, as a standard antibiotic were investigated on inoculated wounds with *Pseudomonas aeruginosa* in Albino mice.

## Experimental

*Animals and animal care*

Twenty Albino male mice, weighing 20-30 gr each, were randomly divided into four equal groups (n = 5), under a 12:12 light: dark cycle with lights on at 06:00 h. Mice were kept under specific pathogenic-free conditions, housed, fed and treated in accordance with the international guidelines principles of laboratory animal use and care (16). They were maintained on standard pellet diet and water ad libitum for 2 weeks to be acclimatized prior to the investigation.

*Anesthesia and wounding*

All mice were anesthetized with 250 µL doses of a Ketamine–Xylazine–saline cocktail (ratio 4:1:35) consisting of Ketamine (Woerden, Holland) 100 mg/Kg and Xylazine (Woerden, Holland) 5 mg/Kg, administered intra peritoneal (17). Briefly, hairs of mice shaved, the exposed skin area cleaned with 70% ethanol, and full-thickness skin wounds (3 mm in diameter) created on the dorsal middle line of mouse using sterile biopsy punch equipment (Revolving punch pliers, Germany). The wounds left open without any dressing material for the duration of the study (18).

*Infected wound model*

The *Pseudomonas aeruginosa* PAO1 obtained from Dr. Nima Jazani, Urmia University of Medical Sciences, kindly. The bacteria were grown in Muller-Hinton broth (Merck, Germany). When bacteria were in the log phase of growth, the suspension centrifuged at 1000 g for 15 min, the supernatant was discarded, and the bacteria were diluted to 10^8^ CFU/mL in sterile Phosphate-Buffered Saline. Ten µl of the bacterial suspension (10^6^ CFU) added to each wound bed immediately after wound surgery (19).

*Grouping and treatment*

As mentioned before the mice were randomly divided into four groups of 5 animals (AgNPs [20 nm, US Research Nanomaterials, Inc. USA], tetracycline, AgNPs along with tetracycline and normal saline as control). In all groups treatments applied topically in the wound bed: 10 µL of AgNPs (0.04 mg/cm^2^) in AgNPs (NP) group, tetracycline (8 mg/Kg) in tetracycline (Tet) group, both of AgNPs (0.02 mg/cm^2^) and tetracycline (4 mg/Kg) in AgNPs along with tetracycline (NP+Tet) group (half of normal dose) and normal saline (10 µL per wound bed) in control group (18-20)

*Measurement of wound infection*

Wound infection was assessed using measurement of bacterial load at the wound site on days 4, 8 and 12 by two methods. First, a swab test was performed from the wound surface for analyzing bacterial superficial load on days 4 and 8. The sample was then transferred to an appropriate diluent. To measure the concentration of bacterial the suspensions were serial diluted from 1:10^3^ to 1:10^12^ with sterile broth media and the dilutions were placed on broth-agar plates. The second test analyzed deep tissue infection on 12 day. The skin was excised as a 3 mm in diameter, including the entire wound with adjacent normal skin.

**Table 1:** Bacterial load average in wounds area of experimental groups post treatment

**Treatment**	**Day**	**Bacterial count**
NP	4	6×10^5^ CFU/10 µL
	8	1.3×10^5^ CFU/10 µL
	12	0 CFU/gr
Tet	4	5×10^5^ CFU/10 µL
	8	1.1×10^5^ CFU/10 µL
	12	0 CFU/gr
NP+ Tet	4	3×10^5^ CFU/10 µLl
	8	6×10^4^ CFU/10 µL
	12	0 CFU/gr
Control	4	1.2×10^10^ CFU/10 µL
	8	3.1×10^9^ CFU/10 µL
	12	6×10 CFU/gr

* Result of Tukey^,^s analysis indicate significant different (P < 0.001) in the between groups of NP, Tet, NP+Tet and control on day 4, day 8 and day 12**.**

**Table 2:** Mean of the wound area on 4, 8 and 12 days in NP, Tet, NP+Tet and control groups. Tet: Tetracycline; NP: Nanoparticle; Tet+NP: Tetracycline along with tetracycline

**Treatment**	**Mean of the wound area (mm** ^2^ **) and (%wound contraction )**			
	**0**	**4**	**8**	**12**
NP Tet NP+Tet Control	7.06	5.48 (22) 5.33 (24) 4.32 (38) 6.21 (12)	2.43 (65) 2.33 (66) 1.50 (78) 3.06 (56)	0.10 (98) 0.04 (99) 0.00 (100) 1.47 (79)

**Figure 1:**
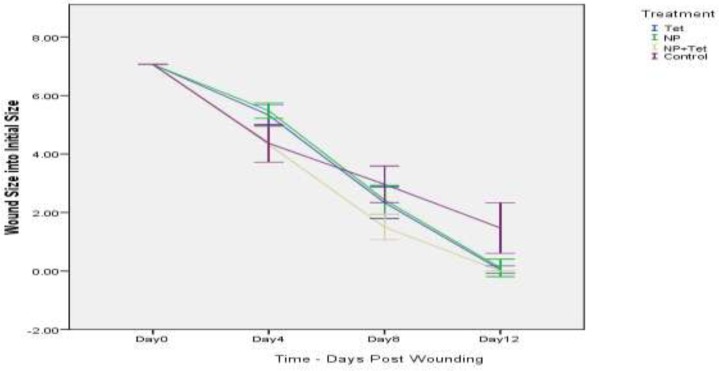
Wound clousre over time. Tet: Tetracycline; NP: Nanoparticle; Tet + NP: Tetracycline along with tetracycline

**Figure 2:**
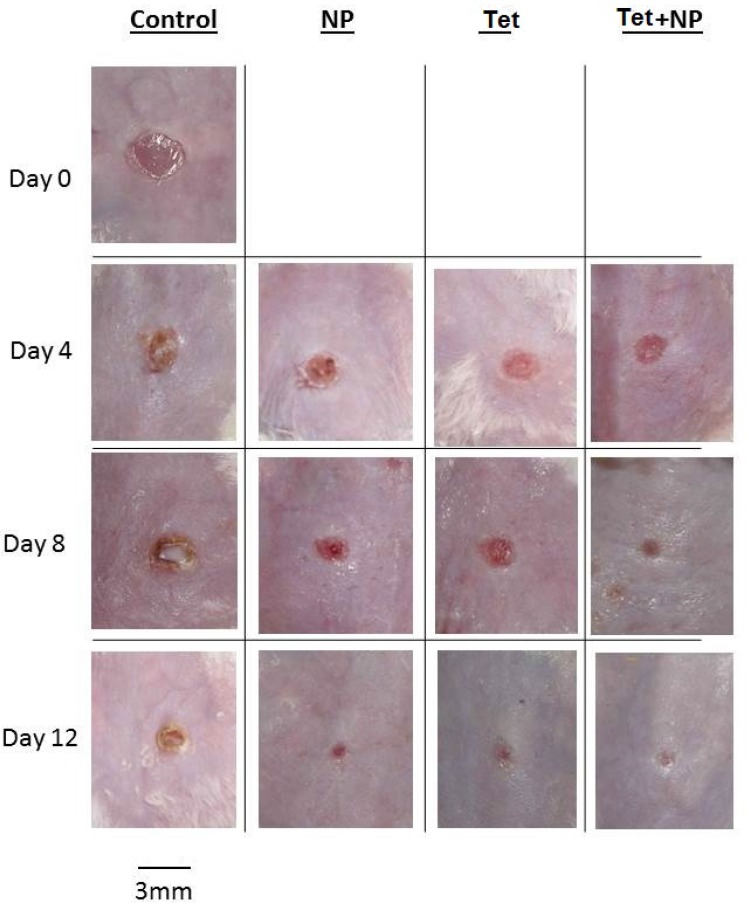
Photograph of wounds in control, NP, NP+Tet and Tet groups on days 0, 4, 8, and 12. Scale bar; 3 mm.

The tissue biopsies were weighed and homogenized in a tissue grinder and placed in 1.5 mL of Muller-Hinton Broth. A drop of the homogenate was placed on the slide and Gram stained for supposal evaluation (The presence of one or more microorganisms per oil-immersion field reflect a microbial load at least 10^5^ CFU/gram). Serial dilutions preparing of using the tissue homogenate 1:10 (0.1+0.9) in the dilution blanks using normal saline. The CFU/gram of tissue was calculated by: CFU/Gram = Plate Count × (1/dilution) × 10/ Wt. of Homogenized Tissue (22-23).

*Measurement of wound size*

Wound healing was monitored by taking digital photographs on days 0, 4, 8 and 12 post treatments. In order to evaluate healing performance, the relic wound size was measured using digital Adobe Photoshop 10 (CS3) software histogram analysis (Parand, Tehran, Iran). Wound size was expressed as the percentage of the wound area determined on every post-treating day, compared with the original wound area: 

%wound contraction = (A_0_ – A_t_)/A_0_ ×100 (14)

Where A_0_ is the original wound area and A_t_ is the area of wound at the time of bacterial counting and taking images (on day 0, 4, 8 and 12 accordingly). Area was measured by the images of the wounds using image analysis software after calibration in same days. All experiment animals were euthanized by CO_2_ inhalation on day 12.

*Statistical analysis*

All statistics performed using SPSS 17.0 (Chicago, IL). Main effect statistical significance was determined at p < 0.05. All results are presented as group means with standard deviation. Statistical analysis was performed using ANOVA analysis.

## Results

*Microbial loads in wounds*

Results of bacterial count in wound surface indicated that except control group, on day 0 to 12, the bacterial loads dropped in all groups. The initial inoculum of approximately 10^6^ CFU/10 µL of organism peaked at about 12×10^9^ CFU/10 µL in the control group on day 8. Some of the wounds in the mice of control group (normal saline) overtly appeared infected (Figure 2). The group of NP+Tet showed the most rapid decrease at the day 0 to day 12 (Figuer 1). The surface infection evaluation showed significant decrease (p < 0.001) in bacterial load in all treatment groups; in NP group, 6×10^5^ CFU/10 µL on day 4 and 13×10^4^ CFU/10 µL on day 8, in Tet group, 5×10^5^ CFU/10 µL on day 4 and 11×10^4^ CFU/10 µL on day 8, in NP+Tet group 3×10^5^ CFU/10 µL on day 4 and 6×10^4^ CFU/10 µL on day 8 (Table 1). By day 12, all of the treated groups showed decreases in deep skin bacterial concentration (0 CFU/gr) compared with control group (6×10 CFU/gr) (Table 1). 

 However, significant decrease (P < 0.001) in deep skin bacterial counts in the AgNPs, tetracycline and AgNPs along with tetracycline were found at any time point compared with control group. Except control group in all treated groups, the wounds did not have infected appearance macroscopically 

(Figure 2).

*Wound closure*

A mouse wound healing model with a 3 mm diameter of full thickness cutaneous wound used to evaluate the effect of the AgNPs, tetracycline, AgNPs along with tetracycline and normal saline (control) on the wound treatment process. Quantitative measurements of wound size are routinely used to assess initial wound size as well as progress toward wound closure. The wound contraction rate measured as the reduction in wound size on days 4, 8, and 12 post treatment. Significant progress in the wound contraction was observed in the treated excision wounds compared with the control group (Table 2). Among all groups, the NP+Tet group demonstrated the best wound healing properties than other groups (Figure 2). The area of wound in NP+Tet group was reduced to 4.32 mm^2^ of their original size (7.06 mm^2^) on day 4, 1.50 mm^2^ on day 8, and 0 mm^2^ (complete closure) on day 12. The corresponding Figures for the control group (normal saline) were 6.21 mm^2^ (day 4), 3.06 mm^2^ (day 8) and 1.47 mm^2^ (day 12). However, the actual wound size was difficult to measure based on digital images, because of the presence of pus in the infected wound in control group. The figures for the standard antibiotic (tetracycline) were 5.33 mm^2^ (day 4), 2.33 mm^2^ (day 8) and 0.04 mm^2^ (day 12), and for AgNPs were 5.48 mm^2^ (day 4), 2.43 mm^2^ (day 8) and 0.10 mm^2^ (day 12). There was complete epithelization in NP+Tet group on day 12 compared with other groups. The tetracycline along with AgNPs achieved 100% wound closure on day 12. In the NP group, the percentage of wound contraction has close figures compared to tetracycline as 98 and 99 percent, respectively (Table 2). However, The images show that most of the treated wounds closed on day 12 (Figure 2). In control group, only 79 percent of the surface area of the wound was closed on day 12 (Table 2).Healing rates among NP+Tet group were significantly faster than NP, Tet and control group (P < 0.001). Result of Tukey^’^s analysis indicate significant differences in the following: Between the groups of NP and control on day 4 (P = 0.002) and on day 12 (P < 0.001); NP and NP + Tet on day 4 (P < 0.001); Tet and NP + Tet on day 4 (P = 0.002); Tet and control on day 4 (P = 0.005) and on day 12 (P < 0.001); NP + Tet and saline control on days 8 (P < 0.001) and 12 (P < 0.001).

## Discussion

The antimicrobial medicines, such as silver compounds are often applied to treat or prevent wound infections. An ideal material not only should be able to protect the wounds against microbial interactions but also should be non-toxic to the human body. The AgNPs at low concentrations have broad spectrum of antibacterial activity; also non-cytotoxic to macrophages at the bactericidal concentration (13).

In the United States, 4 to 6 million people affect by chronic wounds each year, consuming over 25 billion dollars of health-care spending (23). These startling statistics and the health-care risks inherent in the setting of this chronic disease serve as the driving force behind the development of novel wound therapies. Nanomaterials, which either show antimicrobial activity by themselves or elevate the effectiveness and safety of antibiotics administration (24), are called “nanoantibiotics” and their capability of controlling infections *in-vitro* and *in-vivo* has been explored and demonstrated (25). Silver nanoparticles (AgNPs) have been extensively investigated as an antimicrobial agent *in-vitro*. AgNPs show both unique physicochemical properties (high ratio of surface area to mass) and remarkable antimicrobial activities, which confer to them a major advantage for the development of alternative products against, for example, multidrug resistant microorganisms (26). Antimicrobial mechanisms of nanomaterials include: photocatalytic production of reactive oxygen species (ROS) that damage cellular and viral components (27), compromising the bacterial cell wall/membrane, interruption of energy transduction (14), and inhibition of enzyme activity and DNA synthesis (28). Jain *et al.* in 2008 demonstrated that antibiotics-loaded nanoparticles had explored to treat ocular infections by intravitreously administering antimicrobial drugs for sustained medicine release at a high concentration (29). Maya *et al*. demonstrated that bacteria binds and aggregates with Nps increases tetracycline concentrations at the infection site (30). The rapid growth of researches about antimicrobial activities *in-vitro* strengthens the need for closer evaluation of their potential activities *in-vivo*. However, most of the studies were only carried out *in-vitro*, without follow-up of *in-vivo* data support. Here, we describe the use of AgNPs with antibiotic and without antibiotic to improve interactions *in-vivo* on infectious wound healing. Antimicrobial agents, such as antibiotics, silver compounds, iodine compounds, and others are often loaded in wound dressings to prevent or treat infection (31). However, concerns about the using of antimicrobials on open wounds still exist because of their potential cytotoxicity that may delay healing (5). In reviewing the literature, we highlighted the role of AgNPs in the wound healing; this studies have proposed the use of AgNPs as non-infectious wound dressing materials (32). In present study we investigated the role of AgNPs, tetracycline and AgNPs along with tetracycline (in half dose) investigated as an antimicrobial agent for infected wound treatment of mice.

Bacteria are thought to play a critical role in delayed healing by altering host cell function, and lowering the level of endogenous growth factors (33). Strategies to control the risk of infection, and the level of bacterial activity, are generally directed toward several variables – number of bacteria, strength of their virulence, and the immune status of the host (34). The studies showed that AgNPs had the potential to promote wound healing through facilitated anti-inflammatory action (15). The findings provided evidence that AgNPs not only had a beneficial effect on acceleration of the wound-healing process, but also improved the tensile properties of the repaired skin, with a close resemblance to normal skin (35). Mohanty *et al.* in 2012 showed that AgNPs exhibit potent antibacterial activity, besides being non-cytotoxic to macrophages at the bactericidal concentration; also AgNPs represent a potential template for designing of antibacterial agents to target bacterial colonization and to overcome drug resistance (13).

In present study, the hypothesis that the AgNPs and AgNPs along with tetracycline enhance bacterial clearance during wound healing contaminated with *P.aeruginosa* examined. In present study, tetracycline used as reference antibiotic. According to a report by Bucknall in 1980, the rate of infection is directly related to the number of organisms inoculated. Inoculation at 10^6^ bacteria/mL resulted to 100% of the wounds producing pus without mortality while at 10^10^ cells/mL all the test animals died with an overwhelming infection, at 10^4^ cells/mL approximately 50% of the wounds are showed no sign of infection (35). So, in the present study the skin wound of the mice inoculated with 10^6^ CFU of pathogen and a good local infection was established on post-operative days without mortality. The treatment of infectious wounds in mice with AgNPs, tetracycline and AgNPs along with tetracycline resulted in a significant decrease in surface wound infection on days 4 and 8 post treatment. Also significant decrease was cleared in deep wound infection on day 12, suggesting that all of therapies should have positive effects on bacterial wound load. In all three treatment groups the bacterial count reduced from day 0 to 12. 

However, results of wound healing were not statistically significant among NP, Tet and NP+Tet groups but comparing to control group the difference was significant specially on day 12 (almost %19). The difference among NP + Tet and control groups is more significant on day 12 (almost %21). As the AgNPs along with tetracycline were used in half normal dose in NP + Tet group, so it can be stated that they have synergetic effect. 

In Kokura *et al.* study, silver nanoparticles do not readily pass through the skin barrier and have no detrimental effects on skin keratinocytes. They recommend that silver nanoparticles have excellent potential for use as a safe preservative in cosmetics (37). In this study, the presence of silver nanoparticles in both groups (NP and NP + Tet) improved wound appearance better than other groups without silver nanoparticles (Tet and control groups) and wound trace less remains (Figure 2) 

## Conclusion

Finally, present study has described the antibacterial properties and macroscopic wound healing by AgNPs and AgNPs along with tetracycline. The AgNPs and AgNPs along with tetracycline were evaluated as wound dressing materials in infected animal wound models. Application of the dressings showed significant improvement in wound healing macroscopic rate and wound infection treatment. The AgNPs and tetracycline have a synergistic effect together. Application of AgNPs along with tetracycline is more effective than AgNPs and tetracycline alone in reduction of bacterial load and wound macroscopic contraction. These findings support use of the AgNPs along with and standard antibiotic for the treatment of skin infected wounds. Further studies are recommended to study the mechanism of action of AgNPs in infectious wound healing.

*Abbreviations*

AgNPs, Silver nano particles; Tet, Tetracycline, NP, nanoparticles; NP+Tet, Silver nano particles along with Tetracycline. 
